# Developing and Initially Validating the Youth Mental Health Literacy Scale for Ages 11–14

**DOI:** 10.3389/fpsyt.2022.817208

**Published:** 2022-07-15

**Authors:** Joanne Riebschleger, Christine Grové, Kimberly Kelly, Daniel Cavanaugh

**Affiliations:** ^1^School of Social Work, Michigan State University, East Lansing, MI, United States; ^2^Department of Educational Psychology, Monash University, Melbourne, VIC, Australia; ^3^Department of Counseling, Educational Psychology and Special Education, Michigan State University, East Lansing, MI, United States; ^4^School of Nursing and Health Studies, University of Washington, Bothell, WA, United States

**Keywords:** mental health, scale, children, mental health literacy (MHL), youth, families

## Abstract

Despite rising rates of youth mental health disorders and suicides, most youth lack access to accurate, non-stigmatized mental health information. Instead, many describe people with mental illness as violent and incompetent. Mental health literacy aligns with resilience theory. It assumes that youth that have accurate mental health information will have less stigmatized views of mental illness and will be more likely to seek help earlier should mental health symptoms arise. Accurate, non-stigmatized mental health information is especially needed for Children of a Parent or other Family Member that has a mental illness (COPFMI) since they are more likely to acquire a mental illness than children who do not have a family member with a mental illness. COPFMI youth are in need of the same mental health information as general population youth but they can also benefit from knowing how to deal with a family member's mental health disorder. Based on many foundation studies and key stakeholder input from parents, educators, mental health providers, child welfare providers, and especially youth, an emerging Youth Mental Health Literacy (YMHL) scale was developed and validated for measuring the mental health literacy levels of youth ages 11–14. The scale provides a full scale score of youth mental health literacy. It has subscales of knowledge of mental illness and recovery; stigma, help seeking for self/others; coping with stress; and dealing with family mental health challenges. The validation study indicated support for a unidimensional structure for each of the refined subscales. The subscales showed suitable reliability as evaluated by several measures of internal consistency. While the scale needs further study with larger samples of youth, it is hoped that the scale can yield mental health literacy outcome data that can help mental health literacy programs to build evidence-based programs that may, in turn, help prevent, delay, or ameliorate mental health disorders among youth.

## Introduction

The aim of this work is to report an early stage of development and validation of a new scale to measure mental health literacy among youth ages 11–14. The researchers set about to design a scale that could be used to measure mental health literacy (MHL) levels of youth drawn from general population youth and/or those that have a parent or other family member with a mental illness. The idea was to find a scale that could be flexible for use across a range of emerging programs. For example, if a program was delivered to whole classrooms of middle school and high school student general population youth (1), MHL levels could be measured. Program providers targeting youth that have a parent or other family member with a mental illness (2) could also use the scale to measure MHL levels.

Our definition of youth mental health literacy includes all of the constructs/components of mental health literacy, as defined by Jorm (3): *knowledge* of possible mental health symptoms, *recovery* from mental illness, mental illness *stigma*; and *help-seeking* for self and others. This MHL definition was enhanced with the addition of two additional components. We added youth *coping* with stress as a definitional component of mental health literacy (4, 5). The second add-on component of MHL pertains to youth with a parent or other family member with a mental health disorder, specifically their interactions with *family* mental health concerns (6).

The inclusion of youth coping with stress as a part of MHL is based on rapidly rising rates of youth mental health disorders, often preceded and/or accompanied by persistently high levels of youth reported stress (4, 5). Further, a general population mental health literacy program that assessed youth reported stress regularly found youth reported moderate to very high levels of stress throughout the program (6). Since many young people have a family member with a serious mental illness such as a parent and/or sibling, we also included an additional mental health literacy subscale called family, i.e., dealing with family mental health concerns (7).

In this study, youth is defined as ages 11–18; young adulthood includes people ages 19–22. Study participants herein are ages 11–14, aligning with grades 7 and 8th grades, or pre-secondary school enrollment. Mental illness is defined herein as an axis I disorder, as explicated by the American Psychiatric Association (8). Common mental illnesses include anxiety, depression, and substance abuse.

### Youth Mental Health

There are significant risks associated with youth acquiring a mental illness. In the USA, suicide is now the second leading cause of death of people ages 10–34 (9). The CDC revealed a 40% increase in youth reports of feeling persistently sad/hopeless in the pre-COVID time frame of 2009 to 2019. Nearly one in six youth revealed making a suicide plan in the past year, yielding a 44% increase from 2009 to 2019 (10). The World Health Organization or WHO (11) noted, “Mental health disorders account for 16% of the global burden of disease and injury among people ages 10–19” (p. 1). WHO (11) cited the work of Kessler et al. (12) to explain that “half of all mental disorders begin by age 14 but most cases go undetected and untreated” (p. 1). Symptomatic children and adolescents that have not received mental health support or treatment are at risk of substance abuse, violence exposure, poor sexual health, and increased disability-adjusted life years (13). Those youth that continue to experience mental health disorders into adulthood are more likely than adults without a mental health disorder to experience a shortened life span (14), increased physical illness (15), and increased criminal justice involvement (16). People who experience mental illness also face an increased likelihood of being a victim of violent crime (17, 18). They are more likely to experience stigma and discrimination in employment, housing, public services, and interpersonal relationships (19). Suicide and self-harm are particular risks for people with a mental illness (20).

### Children of a Parent or Other Family Member With Mental Illness (COPFMI)

Those at particular risk for acquiring a mental health disorder include children/youth that have a parent or other family member with a mental illness (COPFMI). Nearly 24% of mental health patients in Australian adult services have one or more minor children (21). Campbell et al. (22) reported that many children and adolescents receiving mental health treatment have a parent with a mental illness, specifically 36% of their mothers and 33% of their fathers. This has led to calls for increasing child, parent, and family mental health promotion and prevention programs (3, 23, 24). For example, foundation work conducted by Nicholson (25) recommended support for parents, especially mothers, to reduce parents' mental health symptom levels and developmental risks of their children.

Youth with a parent or other family member with a mental illness often experience family separations, early caregiving, frequent moves, school changes, and high rates of worrying about family and personal well-being (26). Mental health stigma is associated with higher rates of out of home placements for children with a parent with a mental illness (27). Parental mental illness can affect children in many ways, including becoming the caregiver for parents, siblings, and other family members (2). COPFMI have lower levels of academic achievement, social functioning, and school participation (28, 29). Youth living with a parent with a mental illness may experience a challenging family environment that can include parenting difficulties, emotional vulnerability, high stress reactivity, and child feelings of guilt, shame, and loneliness (28, 29). COPFMI are more likely to acquire mental illnesses than children without COPFMI experience (26, 30), especially depression and anxiety.

### Risk and Resiliency

The CDC, as well as WHO, use a risk and resiliency theoretical foundation to consider the process of people acquiring health and mental health disorders. The guiding theory for the YMHL scale development process is similarly based on risk and resiliency, within a frame of youth development. The theory assumes that youth development can be negatively impacted when youth are exposed to risks such as child-parent role reversal, trauma, losses, physical neglect, emotional neglect, bullying, violence, and homelessness. However, risk and resiliency theorist Masten (31) purports that many youth meet, and even exceed, developmental tasks despite exposure to risk factors. Masten (31) says young people can benefit from the application of resilience promoting and ameliorating factors that include coping skills acquisition, social support, community resources, and access to accurate knowledge for dealing with a situational risk. The latter fits especially well with health literacy whereby a patient and/or family members learn about preventing, ameliorating, and/or managing the severity of a health disorder.

### Mental Health Literacy

Mental health literacy is similar to health literacy; individuals and/or family members acquire accurate, non-stigmatized information about a disorder and its treatment (3, 6, 32). Mental health knowledge can contribute toward increased resilience among young people, including those with a parent or other family member with a mental illness (33). Mental health literacy program outcomes can include youth who are more likely to recognize mental health stigma and how to respond to stigma events (7). People with accurate, non-stigmatized mental health information are more likely to seek help earlier for what they suspect may be mental illness symptoms. This is important because the present average delay from symptoms arising to getting mental health services is 8.2 years for those with mood disorder (34). Within this 8.2 year time frame, it took an average of 6.9 years to recognize that a disorder may be present. Thompson et al. (34) also indicated that there is a 1.3 year delay between recognizing the mental health problem, seeking help, and making first treatment contact.

Knowledge of mental illness symptoms could decrease the time span between recognizing disorders and seeking help (1, 35). Bale et al. (32), as well as Riebschleger et al. (7) report that individuals armed with mental health literacy information are more likely to understand that mental illness is common and mental health treatment is usually effective. Those with higher levels of mental health literacy are less likely to hold stigmatized views of mental illness and are more likely to seek help for mental health symptoms of one's self and others (7, 32). Earlier help-seeking among youth could mean that more severe mental illness symptoms and the social and economic secondary effects associated with experiencing mental health stigma may not develop or may be less severe if they do develop. Additionally, earlier help-seeking may reduce feelings of isolation, lower school performance, overall stress levels, and suicidal thoughts (35, 36).

Youth with a parent or other family member with a mental illness that have higher levels of mental health literacy may be likely to state that a family member's level of illness is not their fault. For example, youth are less likely to think about what they should, or should not have done to keep another person in the family from “getting worse” (2, 6, 36). In fact, they are also more likely to report that mental health disorders are health challenges that are nobody's fault. They are more likely to know how to talk about mental illness, recognize that recovery is possible, and state that there is hope for their future. They are more likely to know how to seek help for family and peer mental health crises. They are more likely to know when to help out in the family and when they can relax and “be a kid.” (37).

### Mental Health Literacy Programs

A number of mental health literacy interventions are emerging or developed (38). For example, Mental Health First Aid helps prepare family caregivers and emergency responders to engage in best practices during mental health emergencies (3). Australian programs Be You and Beyond Blue target mental health in early learning services and schools toward building a positive, inclusive, and resilient community (39). Kutcher et al. (1) built a mental health education program for Canadian high school students. Two key purposes are to increase youth ability to seek help earlier for mental health symptoms and to reduce mental health stigma. Family Talk is a program developed by Beardslee et al. (38) that is under development in a number of European countries (40, 41). Family Talk programs help parents with a mental illness to be able to talk to their children about their mental health disorder (41). The idea is to open communication about mental illness among family members. The Youth Education and Support program by Riebschleger et al. (7) especially targets small groups of school youth, some of whom likely have a family member with mental illness. The program delivers 10 activity-based sessions of mental health literacy content to youth ages 11–16.

### A Need for Youth Mental Health Literacy Scales

There are emerging and developed mental health literacy programs that educate about mental health constructs/components (7, 42–44). However, the programs are usually not sufficiently evaluated because of a limited availability of psychometrically valid mental health literacy scales (45–48). Measurement of outcomes of mental health literacy programs will not be able to move toward evidence-based practices until they have access to sound mental health literacy scales. Programs that are not evidence-based face potential barriers to implementation and funding (49).

O'Connor and Casey's (50) review of the literature found that there are few appropriate mental health literacy assessment tools to evaluate mental health literacy programs for young people. They found no available mental health literacy measures covered all aspects of mental health literacy as described by Jorm (3), i.e., knowledge of mental health and recovery; mental illness stigma; and help-seeking/giving. Wei et al. (47) conducted a scoping review of available tools to find that more measures have been developed but most covered only some aspects of MHL and few had undergone strong psychometric validation. Another more in-depth review found that some MHL scales had undergone more rigorous evaluation (48). However, the scales developed for middle and high school students, did not appear to cover all components of MHL (48). Newer scales have been published to evaluate mental health literacy for adults but have too high of a reading level for young people (50, 51). Members of the authors' research team published an earlier Knowledge of Mental Illness and Recovery scale (7) but it did not have new constructs of coping and family.

There is a particular need for general population youth mental health literacy scales that include all of the Jorm (3) mental health literacy components of knowledge of common mental illnesses, recovery from mental illness, mental illness stigma, help seeking for self, and help seeking for others. However, it is important to also measure specific COPFMI ways of dealing with family members' mental illness, such as knowing how to seek help for a family member's mental illness crisis; when to help or not help out at home; and how and when to talk to family members and others about mental health (52). Scale authors can consider including a family component since one of four children may have a parent with a mental illness (21). When siblings and other family members are considered as well, the likelihood of a mental health literacy program participant youth having someone in the family that has a mental illness is even higher. It is also important to consider adding questions on coping with stress as recommended by youth providing input to the development of the scale herein.

There is a need for scales that have reading levels consistent with the reading levels of the youth. For example, for youth ages 11–14, the scale reading level would need to be about ages 9–10 in order to include slower readers. The questions should align with youth-reported mental health perspectives which tend to be behavioral (What do I see people doing and saying?) and not diagnostic (What are the symptoms and diagnoses?) (28). The scale should challenge the youth to consider the accuracy of their cognitive assumptions about mental illness, particularly if they hold stigmatized views of mental illness. Unfortunately, there appear to be limited scales that meet all of the needed criteria, especially for youth ages 11–14.

## Materials and Methods

### A Strong Research Foundation

The Youth Mental Health Literacy Scale content was drawn from many years of research devoted to identifying mental health literacy needs of children and youth. This included research targeting how children and youth with a family member with a mental illness describe their information needs. A wide array of stakeholders informed the scale developers of mental health literacy needs of youth, including children, youth, parents, mental health services providers, child welfare providers, and educators. In addition, authors Riebschleger and Grové spent a good deal of time talking to children and youth about mental illness within the administration of the Youth Education and Support program (6, 53) and a video MHL program for COPFMI youth (2).

Riebschleger et al. (52) surveyed COPFMI experts specifically about what kind of content would be needed for a scale to develop mental health literacy. We then conducted an intensive literature review guided by the overarching research question, “What do children and youth need to know about mental illness?” (36). The data collection included examining literature about the mental health literacy needs of children and youth with a family member with mental illness. Riebschleger et al. are also engaged in developing a youth informed mental health literacy website for adolescents (https://mhiteens.org/). The entire content was generated by youth and young adult suggestions for the recommended content. We also had a youth advisory group that continued to review the mental health literacy content and to make continued suggestions for youth mental health literacy content. The youth suggestions were included in the development of scale constructs.

Across the research projects, we learned: (1) general population *and* COPFMI youth need accurate non-stigmatized mental health information; (2) parents with a mental illness do not often talk to their children about their disorders; (3) children ages 11–16 report experiencing high levels of stress on a regular basis; (4) most children ages 11–16 could identify depressive symptoms but knew little about other disorders; and (5) many children and youth described people with mental illness as looking physically “different”; being violent, dangerous, cognitively incompetent; and unlikely to have “a good life.” Few youth knew the word stigma or its meaning. Some thought people with mental illness were physically ill; several thought they used wheelchairs. Additionally, children with a family member with a mental illness did not seem to know any more about mental illness than general population youth. They knew the most about depression but very few knew about schizophrenia or bipolar disorder. Simply put, youth levels of mental health literacy were low across all of the studies.

### Constructs and Instrumentation

Most of the content needed by COPFMI were the same as general population youth. However, there appeared to be a need to include additional understanding of how to deal with a family member's mental illness, e.g., seeking help for mental illness crises, understanding that mental illness is nothing to be ashamed of, and articulating that mental illness is no one's fault. Many of the COPFMI youth blamed themselves for the symptoms of a person with mental illness (7). The researchers drew on literature and stakeholder needs assessment to determine that a youth mental health literacy scale should cover all of the Jorm (3) constructs, with additional coping and family subscales. Another consideration was that the scale would likely be useful for assessing the outcomes of educational mental health literacy interventions with 11–14 year old middle school youth and for special programs for youth with a family member with a mental illness.

The research team developed items to align with five components: knowledge about mental illness, knowledge of recovery, stigma, help seeking for self, and help for others. These items covered all of the Jorm (3) MHL content areas. Given the rapid rise in stress levels reported by youth and evidence of a connection between stress and the development of anxiety and depression (4, 5), the component “coping with stress” questions was included. In addition, scale questions about living and responding to mental illness behaviors of a family member were developed; they comprise a family subscale. The response options were a closed-format type with a response set of three choices consisting of one correct answer and two incorrect answers, as recommended by a scale development expert. The resultant draft YMHL scale consisted of 74 multiple choice questions with subscales that were given full names followed by abbreviations, i.e., knowledge of MI (K-MI), knowledge of MI recovery (K-R), stigma (S), help seeking for self (HS-S), help seeking for others (HS-O), coping (C), and family subscale (F). The questions were graded correct/incorrect according to an answer key that underwent review by the item developers and the consulting project psychometricians.

The *knowledge* of mental illness subscale combines mental illness and recovery constructs. Subscale question responses focus on mental illness presented as a health disorder that often responds to active treatment. Subscale questions ask about the prevalence of mental illness, common mental illnesses (especially depression, anxiety) and recovery strategies (counseling, medications, social support, healthy habits). Questions identify that mental illness can have a genetic component.

The *stigma* subscale focuses on the discrimination and inaccurate labeling of people with mental illness. Subscale questions ask about people making fun of people with mental illness and experiences of being judged for having a mental illness (assumed incompetent, weak, violent). There is a question about media and social media emphases on mental illness associated with incompetence, weakness, and violence. Some questions focus on the societal tendency to blame people for “causing” the mental illness of selves or others. One question asks about people feeling embarrassed about their mental illness and/or the mental illness of a family member. Other subscale questions report the facts of mental illness: people with mental illness are as intelligent as others in the general population, more likely to be a violence survivor than a perpetrator, able to make decisions, and are usually able to work. People with mental illness can have good lives. They can also be okay parents too (so one should not assume parents with mental illness are neglectful or abusive).

The *help* subscale combines help seeking for self and help giving for others. There is a strong emphasis on talking to adults that may be able to help. There is a specific subscale question on the importance of telling trusted adults at school (and other places) about someone having thoughts of suicide. This is presented as a life threatening situation that youth should not keep secret even if the person might not like them telling. Several questions address how and when to help out at home when a family member has a mental illness and how to support a friend with mental illness.

The *coping* subscale addresses stress management. The questions ask about useful ways to manage stress (talking to someone, exercise, nutrition, relaxation, deep breathing, listening to music, journaling, and positive activities selected by the youth). Negative stress management is described as behaviors that are usually not useful, i.e., yelling at family, friends, and others; breaking things; and using alcohol and illicit drugs.

The *family* scale was built into the instrument to provide flexibility of program evaluation. The family questions are spread across knowledge, stigma, help, and cope subscales. The logic is that family situations can be part of mental health literacy. Professionals delivering MHL programs in general education may or may not want to employ the family subscale as part of their programming evaluation. However, the professionals delivering emerging MHL programs for COPFMI youth are likely to find the family subscale important as one way to measure pre and post intervention outcomes.

### Procedures

Prior to scale administration, we obtained research approval from the Michigan State University Institutional Review Board, and organizational approval from the schools, followed by parent consent, and then youth assent. The draft YMHL scale was administered to youth (*n* = 178) enrolled in biology, gymnastics, and psychology classes located in two middle schools and one high school. The schools were located in a midwestern state. The present research focuses on YMHL scale development for middle school students ages 11-14, and uses data collected from the younger youth (*n* = 85). This validation sample is smaller than the original plan due to the March 2020 school closures in response to the coronavirus pandemic. Co-Vid continues to make collection of data in schools difficult as schools move back and forth between online and in person formats per fluctuating Co-Vid infection rates.

### Analysis

We used a multi-step process to refine the initial item sets for each of the subscales and examine the psychometric properties of the subsequently refined seven subscales using the pilot study data collected from middle school students. Scale refinement was guided by results obtained from item- and subscale-level descriptive statistics, confirmatory factor analysis (CFA), and Mokken Scaling Analysis (MSA), a nonparametric item response theory model (54–56). To evaluate the structural validity of each of the seven subscales, we used both Mokken Scaling Analysis and Rasch modeling (57). Subscale reliabilities were assessed using several internal consistency measures.

Subscale refinement proceeded first with initial item- and scale-level descriptive analyses to assess item scalability and individual youth response quality. Response patterns across all subscale items were examined, and youth with unusual patterns were flagged. Unusual patterns may suggest that individual respondents are not interacting with the instrument in an expected way (i.e., lack of attention, reading level issues) but may indicate that items are not functioning properly. To assess whether the unusual pattern is specific to a respondent rather than to an issue with the item, patterns were examined for multivariate outliers. In the few cases where this was found, respondents with unusual patterns were flagged for later consideration, since later item refinement steps consequently impact the flagged respondent's response pattern. To make a judgment about item functioning, unusual response patterns within an item were also considered. Tetrachoric correlations between item pairs within subscales were used to identify items that did not fit with the remaining subscale items and items with negative correlations were removed from the subscale. All items that all youth got wrong, along with items that all got right, were eliminated, and items exhibiting little variation were flagged for consideration following later refinement steps. Missing data patterns of the youths' responses were examined, and those missing half or more of the responses were removed from the validation sample. Following these steps, the validation sample of each of the subscales consisted of a set of positively correlated items for youth who had data for more than half of the possible number of responses.

Several techniques of MSA were used to further guide item refinement, examine subscale dimensionality, and evaluate response patterns. In contrast to factor analytic techniques, this approach makes few assumptions about the data and does not require large datasets. To flag unusual items or responses in the validation sample, results of Guttman errors and automated item selection procedure (AISP) were examined. Guttman errors are person-specific and used to flag respondents who provide an exceptionally high number of unexpected responses given their responses on remaining items. The unidimensionality of each subscale was examined separately using an automated item selection procedure (AISP) that separates items into like groups, much like what is produced by an exploratory factor analytic approach. The results of this procedure were examined for items inconsistent with a unidimensional scale. The items flagged in the AISP were excluded from subsequent analyses. Following AISP, resultant item sets were tested for local independence, monotonicity, and invariant item ordering. Following further refinement suggested by the tests of these scale properties, the dimensionality of the items and subscales were examined using the homogeneity coefficient (58) typically used with the Mokken scaling approach. The item-specific homogeneity coefficient, an indicator of item scalability, provides a measure of correlation of the item with the remaining items in the subscale. The scale homogeneity coefficient measures the degree to which the total score accurately reflects person ranking on the construct purported to be measured by the subscale. Interpretation of the homogeneity coefficients were guided by rules of thumb developed by Mokken (54).

Factor analytic techniques were also used to explore the dimensional structure of each subscale. Principal components and principal axis factoring were used to explore the number of dimensions for each of the refined subscales. A confirmatory factor analysis model that used the refined subset of items was also estimated for each subscale, and the factor loadings and various model fit statistics were examined to determine the level of agreement between what MSA suggested and what the CFA results indicated regarding the dimensionality of each subscale. The results from the CFA were treated as complementary to the MSA results, rather than as a primary approach to assessing dimensionality due to the strong assumptions of this approach. To estimate a factor analysis model using binary data, one must assume that the binary scores are discretized versions of latent continuous variables, and that the underlying continuous variables have a multivariate normal distribution (59). Because the YMHL is a newly developed scale, we felt that invoking such strong assumptions would be unwarranted at this early stage. Therefore, the MSA results were afforded more weight than the CFA results in subscale refinement decisions. However, because a factor analytic approach is more common in the literature than MSA, we chose to include information about how the scale refinement suggested by MSA compared to that suggested by FA results where relevant.

Items and responses that were flagged by descriptive analyses were noted but retained for use in the MSA and confirmatory factor analysis. These analyses were used as the principal method for informing the refinement of each subscale to a subset of items that work together to measure the dimension of youth mental health literacy targeted by that subscale. The reduced item set for each subscale was then scaled using the Mokken approach, followed by scaling with the alternative approach of Rasch modeling (57) if the data met the strong assumptions of this approach.

Like MSA, the Rasch approach can be used to produce a measure of the construct or latent trait (e.g., knowledge of mental health, stigma) for a respondent. This parametric scaling technique, appropriate for binary items, shares the assumptions of MSA (local independence, monotonicity, and invariant item ordering), but with a more stringent conceptualization of the nature of the underlying construct as a quantitative rather than the ordinal characterization of Mokken scaling. As such, the Rasch approach requires additional stricter assumptions about the distribution of the data in order for the model to be able to make more finely-grained measured distinctions between respondents. Whether the data meet the Rasch modeling assumptions is determined by a number of tests and measures, including model fit and person fit statistics, tests of item infit and outfit, and an assessment of subgroup homogeneity of scores. Assessment of these assumptions proceeds in an iterative manner, as ill-fitting items and persons are removed from the dataset and the reduced dataset to analyzed to detect additional misfitting items and/or persons. A final model is one that is judged to produce acceptable fit statistics with the reduced set of items and persons. The restrictive assumptions of Rasch approach can frequently result in a sizeable deletion of both items and persons and consequently produce a scale with unacceptable reliability (60). In the instances when the data can support a Rasch analysis, the model offers the advantage of more detailed construct measures.

Following scaling, the reliability and the distribution of scores of each subscale were examined. Estimates of the internal consistency of a scale only make sense if the scale is unidimensional; this requirement was assessed prior to determining reliability. Several indices of internal consistency were computed. The accuracy of the usual measure, Cronbach's α, relies on the strict assumptions of Classical Test Theory, which are rarely met in applied contexts. Given this weakness, the estimate is augmented with the calculation of the estimate's bootstrapped 95% confidence interval (61), as well as an additional measure of internal consistency. This alternative internal consistency measure, ω [coefficient omega (62)], relaxes the strict assumptions required by Cronbach's α and has been shown to be a better estimator of reliability (63). Conceptually, ω can be considered as an estimate of the amount of variance of subscale items that is accounted for by a single (unidimensional) latent trait or construct. The values of both α and ω are on the same numerical reliability scale.

Several other descriptive analyses were performed on the refined subscales following scaling. The distribution of the total scores were determined and investigated. The average difficulty and discrimination were estimated for each subscale. The acceptability of the score distribution was considered subjectively as being suitable for separating the youth along the dimensions of MHL. In order for some separation to occur, the scores would need to have enough variation to suggest groupings. Related to meeting this goal, a suitable scale would also produce a score distribution that does not exhibit floor or ceiling effects. The presence of these effects was assessed for the youths' scores resulting from each refined subscale.

## Results

The initial subscale refinement analysis indicated several important findings about the original item sets for each of the subscales. Item-level descriptive statistics were reviewed for each subscale, and these results were used to identify items with substantial overlap that behaved similarly in terms of response patterns. This information, coupled with in-depth review by the item developers reduced the total number of items from 74 to 60. Correlation results suggested that two pairs of subscales could be combined. The Knowledge (K) subscale is a combination of knowledge of MI (K-MI) and knowledge of MI recovery (K-R). The Help-Seeking (HS) subscale is a combination of the help seeking for self (HS-S) and help seeking for others (HS-O) subscales. The items that had negative correlations with the remaining items were removed. Following these refinements, the remaining items were 10 for K, 10 for S, 14 for HS, 13 for C, and 13 for F. The dataset was reduced to include only respondents who provided more than half of the responses for each of the subscales, resulting in a validation data set (*n* = 80) described in Table 1.

**Table 1:** Validation sample descriptives *n* = 80.

	**Percent**
**Gender**	
Female	53.8
Male	42.5
Not reported	3.7
**Race/Ethnicity**	
White	58.8
Asian, Indian, American	
Indian, or Alaskan Native	20.1
Black	5.0
Latinx	3.8
Mixed Race	3.8
Not reported	8.8
**Grade**	
7th	32.5
8th	67.5
	**Mean (sd)**	
Age	12.96 (0.75)

Each subscale was examined for dimensionality using the AISP, and the responses of the youth were assessed for unusual patterns. The AISP analysis indicated that some items within all but the F subscale were not grouping as expected if the subscale were providing a unidimensional measure of the subscale's construct. Using the lowest threshold value for indicating item grouping, item elimination was accomplished iteratively, as items one by one were excluded and the analysis rerun with the reduced item set until AISP results indicated a unidimensional group of items. In making a final decision about elimination of an item, we also considered the results of tests for local independence, monotonicity, and invariant item ordering; all item subsets suggested by the AISP analysis met these tests. The subscale items were also examined with factor analytic models, and the results did not depart from the dimensionality findings using the AISP. Examination of responses using the number of extreme Guttman errors found no more than 10 across the subscales. Given more scale refinement was expected in the scaling step, it was decided to retain high-error respondents and reevaluate their status later in the validation process. Following the refinements given MSA results, the subscales K and H were reduced by 1 item, the S subscale was reduced by 2 items, the C subscale was reduced by 4 items, and the F subscale remained at 13 items.

The structural validity of the five subscales was examined first using MSA. The reduced sets of subscale items were used as the starting set for the MSA scaling procedures. Coefficients of homogeneity for each item, item pair, and the subscale were computed to determine the scalability of each item. Items not exhibiting acceptable levels of homogeneity (minimum threshold of 0.3, as suggested by 52) were excluded from further analyses, and analyses were rerun iteratively after item removal. This was followed by an evaluation of the local independence among items; violating items were removed and local independence was reassessed iteratively until this yielded a set of items that were related through the construct only. Only the S subscale had two items that were flagged at this stage; both were removed. The relationship between the endorsement of items of differing levels of challenge to the measure of the construct was confirmed by evaluating the monotonicity of the scale; all items within each of the subscales conformed with the expected relationship. AISP was rerun to confirm that the resultant item set of each subscale formed a one-dimensional measure. An evaluation of the degree of invariance of the item ordering across different levels of the measure followed. The Guttman errors of respondents were reassessed with the resultant subscale item sets to identify possible idiosyncratic response patterns. The number of respondents exhibiting a high number of Guttman errors ranged from two on the HS subscale to eight on the C subscale. Because the distribution of Guttman errors did not show a concentrated cluster of respondents with high numbers of errors and given the already relatively small sample size, it was decided to retain this small number of youth in the validation sample.

The iterative approach described above was used to produce a final item set for each subscale that did not produce significant violations. After applying the MSA scaling approach, the number of items distributed across the subscales were as follows: a K subscale of nine items, a S subscale of six items, a 13-item subscale for HS, a nine-item C subscale, in addition to a 13-item F subscale measuring the family component across the four domains of knowledge, stigma, help-seeking, and coping. Using the homogeneity coefficient rules of thumb developed by Mokken (54), scalability can be classified as strong, moderate, or weak, based on the inequalities *H* > 0.5, 0.4 ≤ *H* <0.5, and 0.3 ≤ *H* <0.4, respectively. Using these rules of thumb as a guide, the subscales for S, C, and F exhibited strong scalability while the K and HS subscales exhibited moderate scalability. The item scalabilities, item-total correlations, and scalabilities of the subscales are shown in Table 2. Given that all five subscales also met the unidimensionality, monotonicity and local independence assumptions of Mokken scale analysis, this suggests that the sum score for each subscale can be used to reliably order respondents on the construct measured by the subscale.

**Table 2:** Item scalabilities and item-total (point-biserial) correlations.

**Subscale (*items*)**	***H***														
Knowledge (*i* = 9)	0.43	Item	1	11	14	15	22	40	42	60	72				
		*H_*i*_*	0.35	0.92	0.51	0.52	0.47	0.37	0.55	0.29	0.35				
		*r_*bs*_*	0.59	0.61	0.48	0.57	0.70	0.57	0.71	0.53	0.54				
Stigma (*i* = 6)	0.58	Item	25	27	32	48	51	61							
		*H_*i*_*	0.54	0.80	0.72	0.58	0.59	0.42							
		*r_*bs*_*	0.66	0.70	0.70	0.70	0.65	0.62							
Help (*i* = 13)	0.44	Item	35	36	37	45	49	53	55	57	58	59	64	66	67
		*H_*i*_*	0.55	0.39	0.38	0.68	0.42	0.35	0.50	0.37	0.36	0.58	0.45	0.43	0.41
		*r_*bs*_*	0.60	0.57	0.52	0.71	0.60	0.55	0.73	0.58	0.55	0.73	0.68	0.64	0.46
Coping (*i* = 9)	0.69	Item	8	33	34	39	45	51	52	54	55				
		*H_*i*_*	0.70	0.76	0.73	0.73	0.88	0.73	0.56	0.67	0.58				
		*r_*bs*_*	0.57	0.75	0.72	0.63	0.78	0.78	0.66	0.75	0.68				
Family (*i* = 13)	0.53	Item	36	37	38	49	51	53	55	59	62	64	66	68	69
		*H_*i*_*	0.51	0.46	0.84	0.50	0.53	0.43	0.55	0.84	0.90	0.42	0.43	0.51	0.47
		*r_*bs*_*	0.69	0.62	0.75	0.64	0.71	0.59	0.72	0.75	0.80	0.55	0.60	0.59	0.53

Each one of the subscale item sets were then scaled using a Rasch modeling approach. For all the subscales, model and person fit indicators suggested that this parametric scaling approach was not appropriate with the set of subscale items and the sample of youth. An iterative approach to scale refinement using the Rasch model was used, but the results of each subsequent analysis flagged multiple items and persons and produced statistics indicating misfit even after prior misfitting items were removed from the analysis. No suitable subset of items was found to have acceptable Rasch model fit for any of the subscales.

Various characteristics of the subscales, including estimates of internal consistency are shown in Table 3. In addition to the point estimate of Cronbach's α, bootstrapped 95% confidence intervals are provided to give an idea of the level of uncertainty of this point estimate. All confidence intervals include 0.8, the typical reliability target generally desirable for measurement scales. Coefficient ω produced slightly higher reliability estimates and also indicated acceptable reliability given the 95% confidence intervals.

**Table 3:** Subscale descriptives.

**Subscale**	**Initial item set**	**Final item set**	**Score mean (SD)**	**Reliability**		**Difficulty Mean (SD)**	**Discrimination mean (SD)**
				**Cronbach's α** ** 95% *CI***	**McDonald's ω** ** 95% *CI***		
Knowledge	18	9	6.98 (1.92)	0.73 (0.55, 0.86)	0.78 (0.71, 0.90)	0.78 (0.20)	0.59 (0.08)
Stigma	14	6	4.74 (1.47)	0.72 (0.51, 0.84)	0.78 (0.63, 0.91)	0.79 (0.16)	0.67 (0.03)
Help-seeking	16	13	10.99 (2.68)	0.84 (0.70, 0.93)	0.98 (0.83, 1.00)	0.85 (0.10)	0.61 (0.08)
Coping	11	9	7.55 (2.14)	0.85 (0.73, 0.92)	0.89 (0.8, 0.95)	0.84 (0.13)	0.70 (0.07)
Family	22	13	11.16 (2.75)	0.87 (0.71, 0.94)	0.94 (0.68, 1.00)	0.86 (0.09)	0.66 (0.08)

The distribution and descriptive statistics of the subscales show a fair amount of variability among scores. The K and S subscales showed the least score variation, while the HS and F subscales show the most variation in scores. The means and distributions of the scores for all subscales indicate that the youth found the subscales to be a bit on the easy side, and generally showed that the youth were able to correctly answer a majority of the items. The subscales also look similar with respect to the average item difficulty (proportion of respondents correctly answering an item) with all having an average difficulty above 0.77 with moderate variation. The frequency distributions of subscale scores are all negatively skewed, which is consistent with the mean difficulties exhibited by all subscales. A ceiling effect was seen for the C (44%), HS (31%), and F (34%) subscales, while a floor effect is not an issue for any subscale. Item discrimination, the measure of the extent an item can distinguish between low and high scorers, was averaged across the items within a subscale; all subscales showed acceptable item discrimination values.

## Discussion

### Overview

This paper explored the development of a new scale to measure youth mental health literacy in youth ages 11–14. The scale includes all of the Jorm (3) components of knowledge of common mental health disorders, recovery strategies, mental illness stigma, as well as help seeking for self and others in the event of possible mental illness symptoms. This scale could be useful for the general population. We also included coping and family subscales. Since it is possible that children with a family member with a mental illness may comprise almost one of four youth (21), it is likely that the family information will be useful for many within the general population at present and in the future. To our knowledge, this is one of the first youth mental health literacy scales with a reading level appropriate for youth ages 11–14 that covers the full range of mental health literacy components.

The YMHL scale development aligns with calls to develop youth mental health literacy programs (1, 33), particularly given the rising rates of youth mental illness symptoms and suicide (9, 11). The assumptions underlying the need for mental health literacy interventions, and accompanying scales, are that youth can benefit from the application of risk-reducing and resilience-promoting developmental resources. These assumptions are consistent with Masten's (31) risk and resiliency theory. These resources include coping skills, social support, community resources, and access to accurate knowledge for dealing with developmental risks. The latter fits especially well with health literacy aiming to prevent, ameliorate, or manage a health disorder. Standardized scales need to be part of the measurement of the extent of effectiveness of youth mental health programs. The idea is to help move these programs toward becoming evidence-based. Evidence-based programs are more likely to be further funded, tested, revised, and disseminated.

The subscales developed and initially validated provide a means to produce such evidence. The instruments went through a rigorous development process, in which the resultant items were vetted using a variety of psychometric approaches to validate the scales and provide information about the nature of these measures. Using the validation dataset, we found that the five different aspects of youth mental health literacy could be measured with some assurance of the validity of the use of resultant subscales, and with acceptable reliability given our sample data. There is some evidence that the subscales can distinguish between more clustered groups of respondents, but due to the nature of the items and responses, a more finely-grained measure using a Rasch model scaled version of the subscales was not possible.

### Limitations and Strengths

The greatest limitation of this study is a small sample restricted by the onset of the COVID-19 and the need to cut the sample into youth ages 11–14 and youth 15–18 to accommodate developmental knowledge differences. The ages 11–14 scale herein was developed with data drawn from youth attending one of two middle schools in one Midwestern state. Clearly, the scale is newly emerging and requires further testing with more rigorous designs, increased geographic diversity, and especially, larger samples. Larger samples would also allow us to compare results that we obtained using what is likely an inadequate sample size for Mokken scaling analysis and the Rasch model. While we anticipate that Rasch analysis would likely yield the same results with a bigger sample, we would be interested to see if Mokken scaling applied to a larger sample may show more score variability. While the small sample size our results are based on warrants a cautious application of our findings regarding the five subscales, these findings form a basis for continued work on measuring mental health literacy. It is also not known to what extent the scale is useful for particular youth mental health programs so this is an important consideration. The scale needs to align with the YMHL program aims and content.

The greatest strength of the scale lies in its strong foundation studies and stakeholder inclusion. The content was built from findings of numerous needs assessments conducted across many years. Youth input was regularly included in content development. Other stakeholder input was drawn from parents, educators, child welfare professionals, and mental health services providers (2, 6, 7, 36, 64). Family content was drawn from input provided by youth that have a family member with a mental illness, a survey of international researchers in family mental health, and an intensive literacy study intended to flesh out the mental health literacy needs of youth with a family member with a mental illness (36, 37, 52).

### Recommendations

The scale development process leads to recommendations for future research, practice, and policy. Future research should seek a larger sample, more geographically diverse settings, and data should be collected within more rigorous designs including those with control groups and random, or at least wait-list control, sampling. There is likely to be future needs to develop valid and reliable scales for an array of age ranges, e.g., children, young teens, older teens, young adults, and adults. For example, a YMHL scale for ages 15–18 will be forthcoming.

In order to achieve subscales capable of making more finely-grained measures and achieve more detailed distinction of differences in YMHL, future research will also draft and test additional items to be included and vetted in additional validation efforts. The items will aim to provide more variety of challenging questions as well as those targeted at the upper middle of the distribution of YMHL levels. The goal continues to produce an instrument that accurately measures the dimensions of YMHL yet does not present the burden of a large number of additional items.

The authors have requests for access to the YMHL scale from researchers across a number of nations; many of these require translation to a language other than American, Australian or British English (this may also require some translation). It is recommended that the scale translation process be rigorous. The translation of an instrument measuring constructs such as those here presents a formidable challenge for accurate translations. It is well-known and understood that a word for word translation of an instrument is an unacceptable practice. Translation must necessarily be that of the ideas and spirit of each of the items in a contextually accurate manner. The steps of translation must be iterative, and must incorporate a team approach that should include input from the target population of the scale. Furthermore, each translated scale becomes a brand-new scale that must be validated before use.

Recommendations for practice are to continue to develop, test, and revise mental health literacy programs for children, youth, and families. Programs need to continue to acquire and maintain evidence-based practice status. Key stakeholder input is important to further program development processes. Input from parents, educators, mental health, and especially youth, should be gathered and applied to program planning, content, delivery, and evaluation processes. Programs could be delivered in a number of settings but schools may be the best place to access children and youth. Future program development could focus on the specific mental health literacy knowledge needs of diverse groups and cultures. COPFMI mental health literacy programs should be a high priority, especially given youth higher levels of risk for acquiring a mental illness and because many of them live day to day with a family member with a mental illness.

Recommendations for policy are to include mental health literacy programs within the prevention and health promotion arm of a mental health services continuum. Educators, mental health providers, and other community services providers should advocate for funding for mental health literacy programs and research testing. It is even possible that mental health programming would be a regular part of educational programming across all levels of students. That could reduce mental illness stigma and promote earlier help seeking.

## Summary

The Youth Mental Health Literacy Scale for ages 11–14 is designed to measure mental health literacy among general population youth and those with a family member with a mental illness. We will continue to work on refining this scale with larger samples and continued stakeholder input. The scale is intended to yield youth mental health literacy outcome data that can help mental health literacy programs to build evidence-based programs that, in turn, may help prevent, delay, or ameliorate mental health disorders among youth. Perhaps 1 day mental health literacy will be a common health learning activity in schools and communities. It is hoped that the YMHL scale can help play a role toward that shifting paradigm.

## Data Availability Statement

The raw data supporting the conclusions of this article will be made available by the authors, without undue reservation.

## Ethics Statement

The studies involving human participants were reviewed and approved by Michigan State University Institutional Review Board. Written informed consent to participate in this study was provided by the participants' legal guardian/next of kin.

## Author Contributions

JR served as primary investigator for developing the scale based on 12 years of research, wrote scale questions and led the data collection for scale testing, and coordinated the entire manuscript and wrote much of background, part of the methods, and most of the discussion section. CG worked on needs assessment studies and helped write scale questions and wrote part of the background section and edited the manuscript. KK developed statistical findings and wrote most of the methods section and all of the findings section, as well as a small part of the discussion section. DC helped write scale questions and the collect scale testing data and wrote part of the background section and engaged in editing. All authors contributed to the article and approved the submitted version.

## Funding

JR salary was partially supported by the USDA National Institute of Food and Agriculture and Michigan State University AgBioResearch.

## Conflict of Interest

The authors declare that the research was conducted in the absence of any commercial or financial relationships that could be construed as a potential conflict of interest.

## Publisher's Note

All claims expressed in this article are solely those of the authors and do not necessarily represent those of their affiliated organizations, or those of the publisher, the editors and the reviewers. Any product that may be evaluated in this article, or claim that may be made by its manufacturer, is not guaranteed or endorsed by the publisher.
